# Principles of RNA and nucleotide discrimination by the RNA processing enzyme RppH

**DOI:** 10.1093/nar/gkaa024

**Published:** 2020-01-21

**Authors:** Ang Gao, Nikita Vasilyev, Abhishek Kaushik, Wenqian Duan, Alexander Serganov

**Affiliations:** Department of Biochemistry and Molecular Pharmacology, New York University School of Medicine, 550 First Avenue, New York, NY 10016, USA

## Abstract

All enzymes face a challenge of discriminating cognate substrates from similar cellular compounds. Finding a correct substrate is especially difficult for the *Escherichia coli* Nudix hydrolase RppH, which triggers 5′-end-dependent RNA degradation by removing orthophosphate from the 5′-diphosphorylated transcripts. Here we show that RppH binds and slowly hydrolyzes NTPs, NDPs and (p)ppGpp, which each resemble the 5′-end of RNA. A series of X-ray crystal structures of RppH-nucleotide complexes, trapped in conformations either compatible or incompatible with hydrolysis, explain the low reaction rates of mononucleotides and suggest two distinct mechanisms for their hydrolysis. While RppH adopts the same catalytic arrangement with 5′-diphosphorylated nucleotides as with RNA, the enzyme hydrolyzes 5′-triphosphorylated nucleotides by extending the active site with an additional Mg^2+^ cation, which coordinates another reactive nucleophile. Although the average intracellular pH minimizes the hydrolysis of nucleotides by slowing their reaction with RppH, they nevertheless compete with RNA for binding and differentially inhibit the reactivity of RppH with triphosphorylated and diphosphorylated RNAs. Thus, *E. coli* RppH integrates various signals, such as competing non-cognate substrates and a stimulatory protein factor DapF, to achieve the differential degradation of transcripts involved in cellular processes important for the adaptation of bacteria to different growth conditions.

## INTRODUCTION

Accurate substrate recognition is a prerequisite for productive enzymatic function. Many enzymes exploit various structural and chemical peculiarities to ensure discrimination between a cognate substrate and similar compounds from a cellular pool. Other enzymes have evolved to be more promiscuous and recognize multiple substrates that bear common features. Such semi-specific recognition could be challenging for an enzyme if its cognate substrate resembles abundant cellular components.

Widespread hydrolases from the Nudix homology clan break a phosphoanhydride bond in compounds containing a **nu**cleoside **di**phosphate linked to **x** (any moiety) (1) and have to discriminate between cognate substrates and many other abundant cellular compounds that bear diphosphate moieties. Among Nudix enzymes, RppH experiences a particularly significant challenge in recognizing cognate substrates. In *Escherichia coli* and probably many other bacteria, RppH converts diphosphorylated 5′ ends of transcripts into monophosphorylated ends that accelerate subsequent cleavage by the 5′-monophosphate-stimulated endonuclease RNase E (2,3). *Escherichia coli* RppH can also remove pyrophosphate from triphosphorylated RNA with lower efficiency. Thus, the RNA substrate for RppH contains 5′ pp- or 5′ ppp-, chemical moieties present in nucleoside triphosphates (NTPs), nucleoside diphosphates (NDPs) and related compounds, whose millimolar concentrations in cells far exceed those of mRNAs (4). Stressful environmental conditions also elevate the biosynthesis of the alarmone (p)ppGpp to similarly high concentrations (5). All these compounds, in theory, may be suitable substrates for RppH catalysis or at least efficient competitors of RNA substrates in *E. coli* cells.

Does *E. coli* RppH avoid hydrolyzing nucleotides? If not, RppH may deplete the cellular pool of nucleotides and force bacteria to waste energy and resources to restore sufficient levels of nucleotides. Previous *in vitro* studies showed that *E. coli* RppH cleaves off the γ-phosphate of 5′-triphosphorylated mononucleotide substrates at physiological pH and removes both the γ and β phosphates at elevated enzyme concentrations (6). However, under the same conditions, *E. coli* RppH is 10–50-fold more active on 5′-triphosphorylated dinucleotide and trinucleotide RNA substrates, from which it predominantly removes pyrophosphate in a single step. The rate of the enzyme is further increased over 10-fold on diphosphorylated RNA substrates, from which the enzyme cleaves off the β-phosphate (3,6). Furthermore, the metabolic enzyme DapF stimulates *E. coli* RppH activity (7–9) 2–26 fold depending on the composition and length of the RNA substrate (8). Thus, although *E. coli* RppH has the ability to hydrolyze NTPs, such catalysis is slower than with RNA substrates.

The crystal structure of *E. coli* RppH bound to 5′-triphosphorylated trinucleotide RNA provided the first clues as to the molecular basis of discrimination between RNA and nucleotides (6). The structure revealed that RppH specifically binds the 5′ α- and β-phosphates in the catalytic site and recognizes the nucleobase of the second nucleotide of the RNA in a positively charged cleft on the protein surface. The enzyme does not have a specific binding site for the sugar and nucleobase of the first nucleotide. The structure explains biochemical data that revealed the requirement of RppH for two and preferably three unpaired 5′-terminal nucleotides (10), with a purine preferred at the second position because of cation-π stacking interactions and hydrogen bonding to the Hoogsteen edge of the nucleobase (6). The structure suggests that a mononucleotide may bind in the protein cleft but that its 5′ phosphates might have difficulty reaching the catalytic site for hydrolysis. Hence, the lack of extensive binding to the first RNA nucleotide and the large distance between the catalytic site and the binding site for the second nucleotide may constitute the mechanism by which RppH avoids hydrolyzing nucleotides. However, this idea had not been experimentally validated, and the ability of *E. coli* RppH to slowly hydrolyze NTPs had no explanation.

The only data supporting the suggestion that RppH can discriminate against NTPs by binding them in the nucleobase-binding site has come from the crystal structures of an RppH ortholog from *Bacillus subtilis* (BsRppH) (11). These structures demonstrated that the nucleobase of guanosine triphosphate (GTP) binds BsRppH in the site used for recognition of the second RNA nucleotide but that the triphosphate of GTP does not simultaneously bind the catalytic site. In contrast, the crystal structure of *Bdellovibrio bacteriovorus* RppH (BdRppH) showed that the nucleobase of GTP specifically binds the enzyme and that the triphosphate moiety seems to be perfectly positioned for cleavage between the α- and β-phosphates yet does not undergo hydrolysis (12). However, BsRppH and BdRppH are only distantly related to *E. coli* RppH and likely do not use identical catalytic mechanisms (10). Unlike *E. coli* RppH, BsRppH converts 5′-triphosphorylated RNA substrates into monophosphorylated products by releasing the γ and β phosphates in two consecutive steps and strictly requires guanosine at the second position of its RNA substrates (11,13).

To determine how *E. coli* RppH, a representative of the most widespread clade of bacterial RppHs (10), discriminates between cognate and non-cognate substrates and catalyzes the hydrolysis of different substrates, we conducted enzymatic assays and determined a series of X-ray crystal structures of RppH bound to various substrates. Our results suggest that *E. coli* RppH uses distinct mechanisms to bind and hydrolyze different substrates and that competition of RNA with non-cognate substrates shapes the specificity of the enzyme *in vivo*.

## MATERIALS AND METHODS

### Protein preparation

A full-length wild-type *E. coli* DapF and its mutated variant, here called DapF_m_, were prepared as in ref. (8). DapF_m_ contains Y268A and R36A mutations, which disrupt dimerization and abrogate enzymatic activity (14) but do not affect RppH binding and stimulation (7–9). This mutant produces co-crystals with RppH with much higher resolution than the wild-type protein (8). A full-length RppH, and its C-terminal truncated variants, here called RppH_d_ (Δ161-176; protein sequence was Nterm-Ser0-Met1…Gln159-Glu160-Cterm), RppH_s_ (Δ161-176 and K149A/E150A; Nterm-Ser0-Met1…Gln159-Glu160-Cterm) and RppH_t_ (Δ161-176 and Q159A/E160A; Nterm-Ser0-Met1…Ala159-Ala160-Cterm) were used for biochemical and structural experiments as described in the corresponding subsections of ‘Materials and Methods’ section (6,8). The truncated variants did not affect enzymatic activity but yielded better crystals and were predominantly used in crystallization experiments (6,8). RppHs were purified as previously described in refs. (6,8). Briefly, the proteins were produced as protein fusions with N-terminal tandem decahistidine and SUMO tags using T7 RNA polymerase-based expression system and *E. coli* BL21(DE3). The recombinant proteins were purified by affinity chromatography using HisTrap FF column (GE Healthcare) and the tags were cleaved off by the His-tagged ULP1 protease. The cleavage left an extra serine at the N-terminus of all proteins. The tags and protease were removed by HisTrap FF column. RppH was further purified by ion-exchange chromatography on HiTrap SP column (GE Healthcare). Finally, proteins were purified by gel filtration on Superdex 75 (RppH) or Superdex 200 (DapF) (GE Healthcare).

### RNA preparation

RNAs were synthesized by run-off T7 RNA polymerase transcription with DNA templates that comprise a double-stranded T7 promoter and a single-stranded RNA-encoding extension as described in ref. (15). DNA templates were prepared by annealing top and bottom oligonucleotides: TAATACGACTCACTATT (top strand); AGCTAATAGTGAGTCGTATTA (2-mer bottom) and AGACTAATAGTGAGTCGTATTA (3-mer bottom). Transcription was performed in 5 ml at 37°C for 4 h. The total concentration of nucleotides was kept at 15–16 mM, while the concentration of each nucleotide was adjusted according to the RNA sequence. Nucleotides were purchased from Sigma-Aldrich and modified nucleotides were obtained from Axxora. For purification, RNAs were loaded onto a 5-ml HiTrap Q column (GE Healthcare) in 20 mM Tris–HCl, pH 8.0, eluted with a 15–25% gradient of 1 M NaCl in the loading buffer, precipitated with ethanol, washed with 80% (v/v) ethanol, dried and dissolved in water. RNAs were verified by gel electrophoresis, chromatography and mass spectrometry. For MALDI-TOF mass spectrometry, the RNAs were desalted on ZipTip_C18_ tips and eluted in 2,4,6-trihydroxyacetophenone-containing matrix for direct spotting, according to Technical Note 225 (Millipore Corporation).

### Crystallization

RppH was prepared in a solution of 20 mM Na-acetate, pH 5.0, 100 mM NaCl, 1 mM dithiothreitol (DTT) (or 7 mM β-mercaptoethanol). Crystals were grown in hanging drop format typically against 0.4 ml reservoir solution at 20°C for 2–3 days. Crystals of the GTP-bound RppH_s_ were grown from a solution prepared by mixing 2 μl of 0.26 mM RppH_s_ protein and 2 μl of reservoir solution composed of 0.4 M (NH_4_)_2_SO_4_ and 10% (v/v) PEG4000. For soaking, 0.2 μl of 1 mM GTP was added to the drop for several hours. To determine the ppcpG-bound structure, crystals were grown from a solution prepared by mixing 2 μl of 0.26 mM RppH_s_, 2 μl of reservoir solution (0.5 M NH_4_Cl, 10% (v/v) PEG4000) and 0.5 μl crystal seeds obtained for GTP soaking. ppcpG was soaked into crystals by adding 0.2 μl of a solution containing 1 mM ppcpG, 50 mM HEPES-Na, pH 7.5 and 10 mM MgCl_2_. For the ppcpA-bound structure, the 4 μl hanging drop was composed of a premixed solution containing 0.25 mM RppH_t_, 1 mM ppcpA, 0.2 M (NH_4_)_2_SO_4,_ 6.25% (v/v) PEG3350 and 5% (v/v) glycerol. The drop was equilibrated against 0.2 ml reservoir solution composed of 0.4 M (NH_4_)_2_SO_4,_ 12.25% (v/v) PEG3350 and 10% (v/v) glycerol. For cytidine triphosphate (CTP) and uridine triphosphate (UTP)-bound structures, hanging drops were prepared by mixing 2.5 μl 1.2 mM RppH_t_, 20 mM Na-acetate, pH 5.0, 50 mM NaCl and 2.5 μl reservoir solution composed of 0.4 M (NH_4_)_2_SO_4,_ 10% (v/v) PEG3350, 10% (v/v) glycerol. Soaking was conducted by adding 0.2 μl of a solution containing 1 mM CTP or UTP, 0.1 M Na-cacodylate, pH 6.0, 0.2 M Na_2_SO_4_, 15% (v/v) PEG3350 and 10% (v/v) glycerol to the crystal drop and incubating for 30 min. Crystals were cryoprotected by dipping into either reservoir or soaking solution supplemented by either 25% (v/v) glycerol or 25% pentaerythritol propoxylate 5/4 PO/OH as described previously (6).

RppH–DapF complex was formed by mixing DapF_m_ and RppH_t_ at a molar ratio of 1:1.2 in a buffer containing 20 mM Tris–HCl pH 8.0, 500 mM NaCl, and 1 mM DTT. The complex was purified by gel-filtration on Superdex 200 16/600. Crystallization drops of the RppH_t_–DapF_m_ complex were prepared by mixing 0.5 μl of the complex (0.33 mM) with 0.5 μl of reservoir solution containing 30% (v/v) PEG400 and 0.1 M CHES, pH 9.2. Crystals were grown against 80 μl of the reservoir solution in 96-well plates at 18°C for 5–7 days. To soak GTP, GDP or pppGpp, the drops with crystals were supplemented by 0.2 μl solution containing 1 mM nucleotide, 2 mM (for pppGpp) or 20 mM NaF (for GDP and GTP), and 10 mM MgCl_2_ and incubated for 1–4 h. Crystals were cryoprotected in the reservoir solution supplemented with 16% (v/v) glycerol.

### Data collection and structure determination

Diffraction data were collected at 100 K on beamlines 24ID-C of the Advanced Photon Source (Argonne National Laboratory), FMX of the National Synchrotron Light Source-II (Brookhaven National Laboratory) and at the home Rigaku X-ray source. Data were processed using XDS suite (16) or HKL2000 (HKL Research). The crystal structures were solved by molecular replacement using *E. coli* RppH or RppH–DapF complex structures (PDB codes: 6D1V and 4S2Y) as search models and PHENIX (17). The models were adjusted manually in COOT (18) and refined in PHENIX. Organic ligands, water molecules and ions were added at the late stages of refinement based on the *F*_o_-*F*_c_ and 2*F*_o_-*F*_c_ electron density maps. Occupancy of some ligands was refined based on the residual density map.

### TLC analysis of RppH activity

Reactions for thin layer chromatography (TLC) were performed with 1 mM nucleotide substrates and 5 μM of the full-length RppH, in a 10-μl solution containing 10 mM MgCl_2_ and 50 mM buffer with different pH. Buffers were: MES-NaOH, pH 6.0; Na-cacodylate-HCl, pH 6.5; MOPS-NaOH, pH 7.0; HEPES-NaOH, pH 7.5; Tris-HCl, pH 8.0, 8.5 and 9.0. Reactions were incubated at 37°C for 1 h and quenched with 5 μl of 150 mM ethylenediaminetetraacetic acid (EDTA). Samples were analyzed on PEI-cellulose plates (F-254, Selecto Scientific) in 0.3 M potassium phosphate buffer, pH 7.5, for adenosine triphosphate (ATP), adenosine diphosphate (ADP), GTP, GDP, pppAG (RNA), ppGpp and pppGpp, and pH 3.3 for CTP and CDP, UTP and UDP. RNAs were visualized by UV_254_ shadowing. The equimolar amount of wild-type *E. coli* DapF (8) was added to the reactions for testing RppH activity stimulation at pH 7.5. Inhibition of RppH activity was conducted using NaF (Sigma-Aldrich) at 1–10 mM. For TLC experiments, original colored images were converted to black-and-white images, and the levels of brightness were adjusted to enhance the contrast and better visualize reaction products.

### Kinetics of RppH reactivity monitored by chromatography

RppH activity on pppAG and ppAG RNAs, NTPs, NDPs, and ppGpp was determined by kinetic analysis, as described in ref. (6). Each reaction mixture contained 0.1 mM substrate and 1 μM full-length RppH in case of nucleotides and 0.1 μM RppH in case of RNA, in a 50-μL solution containing 50 mM HEPES-NaOH, pH 7.5, 10 mM MgCl_2_ , 0.1% Triton X-100, at 37°C. Reactions were incubated for 0–20 min and quenched with 750 μl of 50 mM sodium acetate, pH 4.5. The quenched reaction samples were analyzed by anion-exchange chromatography on a 5 × 50 mm MonoQ column (GE Healthcare). The reaction products and remaining substrates were eluted with a 0–1.0 M NaCl gradient in 10 mM Tris-HCl, pH 7.5, detected at 260 nm, and the amount of each mono- or oligonucleotide was calculated by using the integration function in UNICORN software (GE Healthcare). The initial reaction rates were calculated based on the ratio between remaining substrate and reaction products using a linear fit model of GraphPad. Each assay was repeated at least twice.

Competition experiments were conducted using the same protocol as cleavage assays with the 0.1 mM substrate RNA concentration, the nucleotide concentration ranging between 0.1-5.0 mM, and 10 min incubation. The initial reaction rates were calculated based on the ratio between reaction products and remaining substrate using a linear fit model of GraphPad software. Because of peak overlap, inhibition of GTP hydrolysis was determined solely based on quantification of the reaction products. IC_50_ values were determined using an IC_50_ model of GraphPad.

### RppH reactivity monitored by pyrophosphate assay

Inorganic pyrophosphate assay was performed colorimetrically according to the published method (19,20). Each reaction mixture contained 500 μM pppApG RNA and 1 μM RppH_t_ in a 20-μL solution containing 10 mM MgCl_2_, 0.1% Triton X-100 and 50 mM buffer. Buffers were: acetic acid-NaOH, pH 5.0 and 5.5; MES-NaOH, pH 6.0; Na-cacodylate-HCl, pH 6.5; MOPS-NaOH, pH 7.0; HEPES-NaOH, pH 7.5; Tris-HCl, pH 8.0, 8.5, and 9.0. Reactions were quenched with 80 μL of 1% SDS after 1 min incubation at 37 °C. Inorganic pyrophosphate from the quenched reactions was precipitated in the presence of 1 mM CaCl_2_ and 100 mM NaF. Precipitate collected by centrifugation at 20,000 × g for 10 min was washed with acetone, dried, and re-dissolved in solution containing 1.25 N H_2_SO_4_, 10 mM ammonium heptamolybdate and 40 mM β-mercaptoethanol. Intensity of green color developed after 1 h incubation at room temperature was measured at 700 nm and the amount of the pyrophosphate liberated during reaction was calculated from the calibration curve prepared with standard solutions of sodium pyrophosphate.

### Metal dependence of RppH reactivity

Reactions were performed with 5 mM GTP and 5 μM RppH_d_, in a 20-μL solution containing 10 mM divalent cation salt (MgCl_2_, MnCl_2_, NiCl_2_, or CaCl_2_) and 50 mM buffer with different pH (HEPES-Na, pH 7.5; Tris-HCl, pH 8.0, 8.5, 9.0). Reactions were incubated at 37 °C for 1 h and quenched with 10 μL 150 mM EDTA. Samples were analyzed on PEI-cellulose plates in 0.3 M potassium phosphate buffer, pH 7.5, at room temperature. RNAs were visualized by UV_254_ shadowing.

### Determination of ATP concentration

WT, Δ*rppH* and Δ*rppH*+pPlacRppH strains were from (21). Fresh colonies were picked up from the agar-LB plates and grown in 0.5-2 mL LB medium without antibiotics at 37°C for about 4 h until A_600_ reaches ∼1.0. Bacteria were diluted ∼100-fold in LB medium without antibiotics and incubation continued until A_600_ reached 1.0 for WT, Δ*rppH* and Δ*rppH*+pPlacRppH (-IPTG) cultures. Δ*rppH*+pPlacRppH (+IPTG) and Δ*rppH*+pPlacRppH (+10 × IPTG) cultures were induced by 10 and 100 μM isopropyl β-D-1-thiogalactopyranoside (IPTG), respectively, at A_600_ ≈0.5 and incubation continued up to A_600_ ≈1.0. 3 ml aliquots of cell cultures were collected by centrifugation and resuspended in 1 ml 20 mM Tris–HCl, pH 8.0, 150 mM NaCl buffer. Cells were centrifuged again and resuspended in 600 μl buffer. ATP concentration was measured by Luminescent ATP Detection Assay Kit (ab113849, Abcam). Briefly, 100 μl cells were lysed by 50 μl Detergent Solution by shaking in an orbital shaker at 600–700 rpm in a 96-well plate (No. 655209, Greiner Bio-One) at room temperature for 5 min. The 50 μl Substrate Solution was added to the well and shaking continued for 5 more min. The plate was placed in the dark for 10 min and luminescence was measured immediately at room temperature using FlexStation 3 Multi-Mode Microplate Reader (Molecular Devices). The luminescence counts were determined as averages of three–four technical replicates. The final values were determined by averaging data from three biological replicates.

## RESULTS

### RppH hydrolyses NTPs and NDPs with low efficiency at intracellular pH

At pH 7.5, *E. coli* RppH has the ability to hydrolyze NTPs, which mimic the 5′-triphosphorylated end of RNA (6). However, RppH removes orthophosphate from 5′-diphosphorylated RNAs at least 10 times faster than it removes pyrophosphate from their triphosphorylated counterparts and likely uses 5′-diphosphorylated RNAs as substrates in *E. coli* cells (2,3). If a diphosphorylated RNA 5′-end is a better substrate for RppH, are NDPs better substrates for RppH than NTPs?

**Figure 1.**
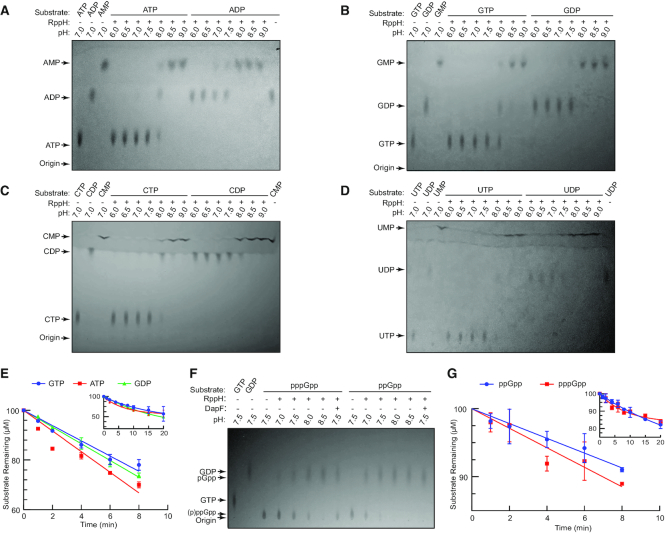
Hydrolysis of the 5′-phosphorylated ends of nucleotides by *Escherichia coli* RppH. (**A**–**D**) Removal of 5′ phosphates from ATP and ADP (A), GTP and GDP (B), CTP and CDP (C), and UTP and UDP (D) at different pHs. The cleavage products were analyzed by TLC. (**E**) Kinetics of hydrolysis with GTP, ATP and GDP. To calculate the initial rates of the reactions, the data from early time points (0–8 min) were fit to a linear regression model (*n* = 2, error bars represent the standard deviation (SD)). The inset includes data for longer incubation times. The cleavage products were analyzed by ion-exchange chromatography. (**F**) Hydrolysis of the 5′ ends of pppGpp and ppGpp at different pHs, with and without *E. coli* DapF, as analyzed by TLC. (**G**) Kinetics of hydrolysis on pppGpp and ppGpp. The data were fit as described for panel (E).

To compare the activity of *E. coli* RppH on NTPs and NDPs, we incubated RppH with nucleotides and 5 mM Mg^2+^ over a wide pH range at 37°C and analyzed the results by TLC (Figure 1 and Supplementary Figure S1). Although the cytoplasmic pH in *E. coli* cells is maintained between 7.1 and 7.8 (22,23), changes in external pH can shift the intracellular pH out of this range temporarily (23–25).

The TLC results showed that in the absence of RppH, neither nucleotide was hydrolyzed spontaneously at all tested pHs (Supplementary Figure S1A–D). RppH did not hydrolyze any of the four NTPs at acidic pH and minimally cleaved off orthophosphate at pH 7.0 and 7.5 (Figure 1A–D). At pH 8.0, the enzymatic activity increased substantially, converting about two-thirds of the substrates to roughly similar amounts of NDPs and NMPs. At pH 8.5 and 9.0, all of the ATP and a majority of the GTP, CTP and UTP were converted to NMPs, with a small amount of CDP and UDP left undigested.

Similarly, NDPs remained largely intact at pH 6.0 and 6.5. Weak hydrolysis was observed at pH 7.0 and 7.5, and all of the ADP and almost all of the GDP, CDP and UDP were hydrolyzed at pH 8.0. At higher pH, RppH hydrolyzed all substrates. Thus, RppH has a low activity on NTP and NDP substrates at pH values (7.0–7.5) that are comparable to the intracellular pH. However, the enzyme is more reactive at pH 8.0, a value that approaches the upper boundary of the stably maintained intracellular pH. At least at this pH, RppH hydrolyzes NDPs better than NTPs, revealing that the pH dependence of NDP hydrolysis relative to NTP hydrolysis is shifted somewhat toward neutral pH.

To compare the initial rates of the reaction with NTPs and NDPs as substrates, we determined the kinetics of hydrolysis for GTP, ATP and GDP at pH 7.5 by using ion-exchange chromatography. To slow the reactions, we used only one-fifth as much RppH. Under these conditions, only 20–30% of nucleotides were hydrolyzed during a 10 min incubation (Figure 1E). Longer incubation with GTP and ATP resulted in reactions approaching a plateau at which ∼70% of undigested substrate remained, while the reaction with GDP proceeded further to ∼50% conversion, without approaching a plateau. The initial reaction rates, calculated using linear regression, were similar for all three nucleotide substrates. At pH 7.5, ATP was a slightly better substrate (4.2 ± 0.24 μM/min, *n* = 2, error is SE) than GDP (3.4 ± 0.07 μM/min) and GTP (3.1 ± 0.13 μM/min). As expected, under the same conditions, the reaction rates of the RNA substrates were over 10 times faster than NTP hydrolysis (Supplementary Figure S1G).

To determine whether DapF can stimulate the reactivity of RppH with nucleotide substrates, we conducted reactions with NTPs and NDPs in the absence and presence of DapF at pH 7.0, 7.5 and 8.0. The TLC results showed that DapF only slightly accelerated these reactions (Supplementary Figure S2).

### RppH is most active in the presence of Mg^2+^ cations

Although *E. coli* RppH and its homologs react with RNA most efficiently in the presence of Mg^2+^ cations (6,26–31), some Nudix proteins use Mn^2+^ and other divalent cations for catalytic activity (32,33). To determine which cations are most effective at stimulating the reaction of RppH with nucleotide substrates, we compared *E. coli* RppH activity on GTP in the presence of Mg^2+^, Mn^2+^, Ni^2+^ and Ca^2+^ at pH values ranging from 7.5 to 9.0 (Supplementary Figure S3). The TLC data revealed that RppH removes orthophosphate or pyrophosphate from GTP most efficiently in the presence of Mg^2+^ cations. RppH also displays some activity in the presence of Mn^2+^ cations but no activity with Ni^2+^ or Ca^2+^ cations.

### RppH hydrolyses pppGpp and ppGpp with low efficiency

Amino acid starvation and other stresses elevate concentrations of the cellular alarmone (p)ppGpp to millimolar levels (5,34). (p)ppGpp binds to many *E. coli* proteins, including Nudix hydrolases (35), and it is conceivable that its 3′ phosphates could enable it to bind RppH more strongly than GTP by interacting with the positively charged surface of the enzyme. Our TLC experiments revealed very slow hydrolysis of pppGpp at pH 7.5 and moderate hydrolysis at pH 8.0 (Figure 1F). As observed for GDP versus GTP, the pH dependence of ppGpp hydrolysis was slightly shifted toward neutral pH and slow hydrolysis was detected even at pH 7.0. About half of ppGpp was hydrolyzed at pH 7.5 and most ppGpp was hydrolyzed at pH 8.0. DapF slightly accelerated the hydrolysis of both nucleotides. In the absence of RppH, neither nucleotide was hydrolyzed (Supplementary Figure S1E).

The kinetics of hydrolysis determined by ion-exchange chromatography revealed initial rate of 1.20 ± 0.07 and 1.30 ± 0.10 μM/min for ppGpp and pppGpp, respectively, at pH 7.5 (Figure 1G). These rates were similar to the rates of NTP and NDP hydrolysis, although ppGpp and pppGpp were ∼2.8 and ∼2.4 times less reactive than GDP and GTP, respectively.

### RppH has different pH optima for RNA and nucleotides

**Figure 2.**
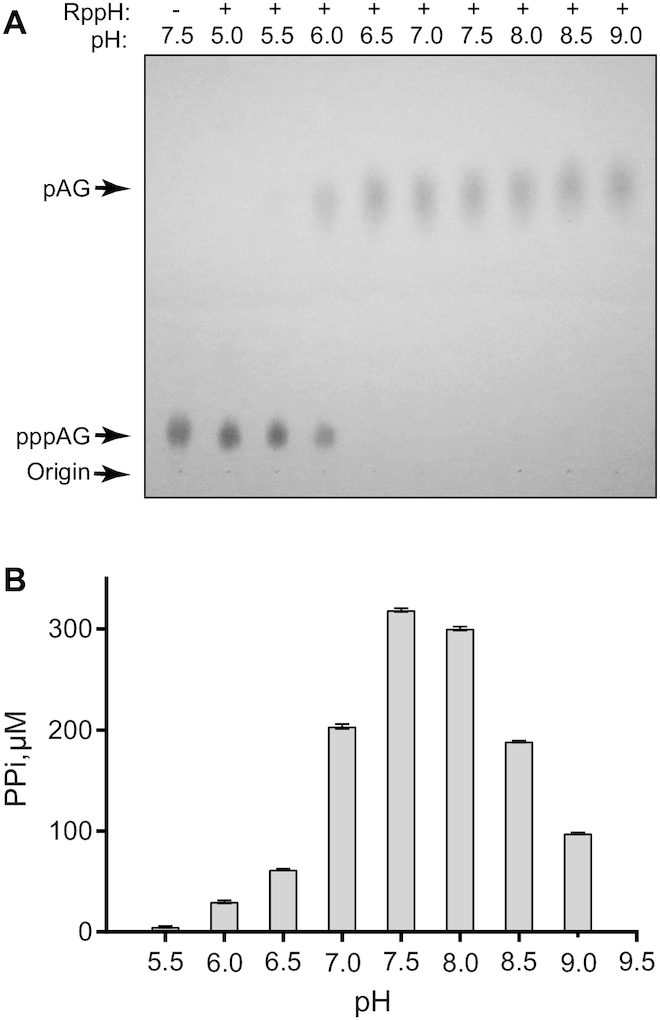
pH dependence of pyrophosphate cleavage from the triphosphorylated 5′ end of pppAG RNA by *Escherichia coli* RppH. (**A**) The cleavage products were analyzed by TLC. (**B**) RppH reactivity monitored by assaying for pyrophosphate (PPi) production (*n* = 2, mean ± standard error (SE)).

The higher reactivity of nucleotides with *E. coli* RppH at pH ≥ 8.0 led us to investigate the optimal pH for the hydrolysis of RNA substrates. To answer this question, we tested the activity of RppH on the RNA dinucleotide pppAG. TLC experiments revealed that RppH activity could be detected at a pH as low as 5.5 and that hydrolysis is most efficient at pH ≥6.5 (Figure 2A). In the absence of RppH, the RNA ends were not hydrolyzed (Supplementary Figure S1F). To determine the optimal pH for the reaction, we performed quantitative molybdate assays to measure the initial rates of pyrophosphate release from the RNA (Figure 2B). In contrast to the reaction with nucleotides, this assay revealed a pH optimum of 7.5 for the reaction of RppH with RNA. At pH 7.0, its reactivity with RNA was only 1.6 fold lower. Thus, the reactivity of RppH with its cognate substrate, RNA, is maximal at intracellular pHs, while its reactivity with non-cognate substrates, nucleotides, is optimal at more alkaline pHs.

### Nucleotides and alarmones inhibit RppH activity *in vitro*

**Figure 3.**
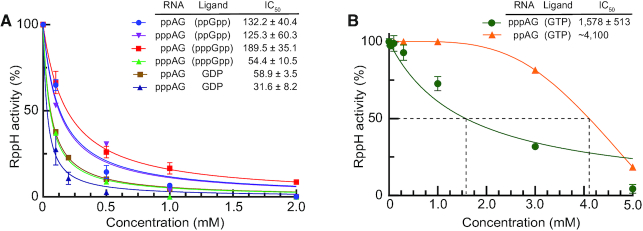
Inhibition of RNA hydrolysis by nucleotides. (**A**) Reactivity of RppH with ppAG and pppAG RNAs in the presence of the nucleotides indicated in parentheses. IC_50_ values (*n* = 2, mean ± SE, μM) were calculated by fitting the data to the inhibition model. Error bars are SD. (**B**) Inhibition of RppH reactivity by GTP. The reaction with ppAG does not fit the inhibition model well, and therefore the IC_50_ values were estimated from a smooth curve connecting the data points.

To determine whether nucleotides can compete with RNA substrates and negatively affect the reaction rate of RNA with RppH, we monitored the reaction of RppH with the dinucleotide RNAs pppAG and ppAG, in the presence of 0.1–5.0 mM concentrations of the guanosine nucleotides GTP, ppGpp and GDP and 10 mM Mg^2+^ (Figure 3). Competition with ATP could not be accurately quantified because of overlap between the peaks.

Although the nucleotides were not detectably hydrolyzed under these reaction conditions, they had profound inhibitory effects on the reaction rates of the dinucleotide RNA substrates. GDP was the most efficient inhibitor, with an IC_50_ of ∼32 and 59 μM for pppAG and ppAG, respectively (Figure 3A). ppGpp and pppGpp inhibited the reactions less efficiently, with IC_50_ values in the range from 54 to 190 μM. GTP was the least efficient competitor, requiring concentrations of ∼1.6 and ∼4.1 mM to achieve 2-fold inhibition of the reaction of RppH with pppAG and ppAG, respectively (Figure 3B). Interestingly, higher concentrations of GDP, pppGpp and GTP were required to inhibit the reaction of RppH with the diphosphorylated RNA than with the triphosphorylated RNA. Since the inhibitory concentrations of NTPs and NDPs are comparable with their intracellular concentrations while stresses surge (p)ppGpp concentrations over their inhibitory values (see ‘Discussion’ section for details) (4,5), cellular nucleotides may noticeably and differentially inhibit the reaction of RppH with various RNA substrates in bacterial cells.

### NTPs bind RppH in the RNA-binding cleft

**Figure 4.**
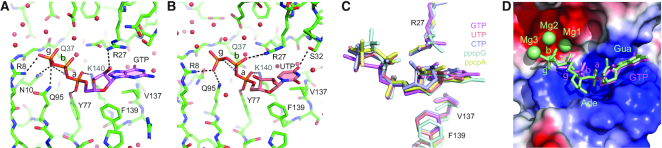
Crystal structures of NTPs bound to *Escherichia coli* RppH. (**A** and **B**) Zoomed-in views of GTP- (violet) and UTP (salmon) -bound RppH structures. RppH and NTPs are shown in atomic colors (red, oxygen; blue, nitrogen; orange, phosphorus; green, salmon or violet, carbon). Putative hydrogen bonds are shown as dashed lines. Water molecules are red spheres. Phosphates are indicated with Greek characters. (**C**) Superposition of NTPs and their non-hydrolyzable analogs from the structures of NTP–RppH complexes. All-atom superposition was centered on the entire RppH–NTP structure. (**D**) Superposition of GTP (violet sticks) and ppcpAGU RNA trapped in a catalytically competent conformation (green sticks) from the structures of RppH–bound complexes determined in the current study and ref. (6), respectively. The surface of RppH is shown in electrostatic-potential presentation. Mg^2+^ cations found in the RNA-bound structure are shown as green spheres. Ade, adenosine; Gua, guanosine.

To understand how nucleotides inhibit the reaction of RppH with the RNA substrates, we determined 1.6–1.9 Å crystal structures of RppH bound to triphosphorylated nucleosides and/or their non-hydrolysable analogs, ppcpNs, which contain a methylene moiety instead of a bridging oxygen atom between α- and β-phosphates (Supplementary Table S1, Figure 4A-B and Supplementary Figure S4A-B). Neither structure contains Mg^2+^ cations since their addition to the crystallization solution resulted in the loss of electron density map for nucleotides, even for non-hydrolysable substrate analogs and at pH values unfavorable for catalysis. In the absence of Mg^2+^, the map for nucleotides was of high quality although the occupancy of nucleotides was refined to lower than 100% in some cases (Supplementary Figure S4C–F).

In all of the structures, the position of the nucleobases is nearly invariant, while the conformations of the sugar–phosphate moieties have small differences (Figure 4C). All of the nucleobases bind in the protein cleft where we previously observed specific binding of guanosine at the second position of an RNA ligand (Figure 4D). In this cleft, the nucleobase is sandwiched between the guanidinium group of R27 and a hydrophobic pedestal formed by V137 and F139 (Figure 4C). Pyrimidines are locked in place by a hydrogen bond with S32 (Figure 4B and Supplementary Figure S4A), while purines are likely held by stronger cation–π interactions with R27 (Figure 4A and Supplementary Figure S4B). The sugar–phosphate moiety is oriented toward the catalytic site and forms several hydrogen bonds involving R27, Y77, Q95 and K140 in all of the structures.

Superposition of the RNA- and GTP-bound structures shows that the triphosphate moiety of GTP does not reach the catalytic site because the mononucleotide is shorter than an RNA dinucleotide (Figure 4D). In contrast, the additional nucleotide in the RNA dinucleotide helps it to span the distance between the cleft, where the nucleobase binds, and the catalytic site, where the triphosphate is positioned. The superposition shows that the α-,β-and γ-phosphates of GTP are positioned in place of the phosphate that connects the first and second nucleotides of the RNA, the sugar of the first nucleotide, and the 5′-terminal α-phosphate, respectively. To reach the catalytic Mg^2+^ cations, NTPs have to slide toward the active site by one phosphate distance, effectively removing the nucleobase from the cleft and placing it into a less favorable binding site. Thus, the NTP-bound structures demonstrate that NTPs interact with RppH in the same site where the second nucleotide of RNA binds RppH. In this position, NTPs can compete with RNA for RppH binding and inhibit the reaction of RNA substrates.

### pppGpp hydrolysis requires an additional Mg^2+^ cation

The NTP-bound structures, determined in the absence of Mg^2+^ cations, did not explain how RppH catalyzes the hydrolysis of NTPs, NDPs and (p)ppGpp, which have two or three 5′-terminal phosphates. To trap the enzyme–substrate complexes in the catalytically relevant pre-cleavage conformation, we used an alternative approach. First, we used crystals of the RppH-DapF complex (8). These crystals proved to be more robust at alkaline pH values than crystals of RppH alone, and thus are better suited for trapping the enzyme–substrate complex in an active conformation. Second, we soaked ligands into these crystals in the presence of Mg^2+^ to ensure binding of the phosphorylated 5′ end and fluoride anions to inhibit RppH-catalyzed hydrolysis by replacing the reactive water molecule or hydroxide ion (Supplementary Figure S5A) (32,36–37).

In this manner, structures were determined for RppH bound to pppGpp, GTP and GDP (Supplementary Table S2). All three maps had electron densities corresponding to the bound ligand and additional densities for metal ions and water molecules in the catalytic site. Soaking the crystals with other nucleotides or ppGpp did not yield electron density for bound ligands.

**Figure 5.**
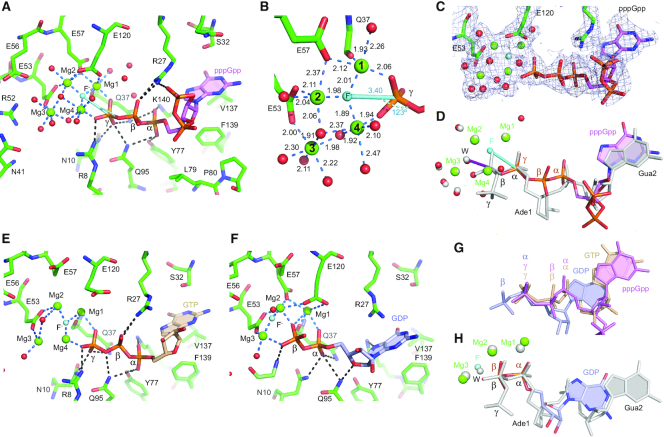
Crystal structure of pppGpp, GTP and GDP bound to RppH in the presence of Mg^2+^ and F^−^. (**A**) A view of pppGpp (violet) bound to RppH (green). Mg^2+^ cations and water molecules are shown as green and red spheres, respectively. A putative F^−^ anion is depicted as a light blue sphere. In-line attack on the γ-phosphorus atom is depicted as a light blue stick. Coordination and hydrogen bonds are shown as dashed blue or black lines, respectively. (**B**) Zoomed-in view of the catalytic site. Coordination distances are indicated in angstroms. Note the position of the F^−^ ion (replacing the reactive water molecule) between Mg1 and Mg4; when it attacks the γ-phosphorus atom of RNA, the reactive water molecule is located between Mg2 and Mg3. (**C**) Composite simulated annealing omit map (1 σ level) shown with the refined structure. (**D**) A superposed view of pppGpp (colored) and ppcpAGU RNA (gray) bound to RppH in structures from the current work and ref. (6), respectively. Ade, adenosine; Gua, guanosine. Note the additional Mg^2+^ cation Mg4 bound to pppGpp. In-line attack on the β-phosphorus atom of RNA is depicted as a purple stick. (**E** and **F**) Views of RppH-bound GTP (light orange) and GDP (light blue). (**G**) All-atom superposition of pppGpp, GTP, and GDP bound to RppH. (**H**) All-atom superposition of GDP and ppcpAGU RNA bound to RppH in structures from the current work and ref. (6), respectively.

The resolution of the structure with bound pppGpp was the highest (2.05 Å) (Figure 5A). As in the NTP-bound structures determined in the absence of Mg^2+^ cations, the nucleobase of the ligand sits in the protein cleft while the triphosphorylated 5′ end extends toward the catalytic site. All three 5′-terminal phosphates make hydrogen bonds with amino acids of RppH, including R8, N10, R27, Q37, Y77, Q95 and K140. While only one residue, R27, contacts the β-phosphate, four near the catalytic site hold the γ-phosphate, whose position is most critical for catalysis. The diphosphorylated 3′ end does not interact with RppH extensively, with only the terminal phosphate forming a hydrogen bond with the side chain of R27.

In the complex with pppGpp, the catalytic site contains four Mg^2+^ cations coordinated with octahedral geometry by several amino acids and water molecules (Figure 5A–C). Mg1, Mg2 and Mg3 bind to two residues of the Nudix motif, E53 and E57, as well as to Q37 and E120. The fourth cation, Mg4, does not bind to the protein and instead is coordinated by the non-bridging oxygen atom of the γ-phosphate of pppGpp and by water molecules, which are additionally coordinated to Mg1, Mg2 and Mg3. Since F^−^ and water molecules cannot be distinguished using electron density maps at this resolution, we assigned one density peak, between Mg1 and Mg4, to F^−^ on the basis of coordination geometry and distances (Figure 5B), as well as previously determined structures of F^−^-bound inorganic pyrophosphatase (38). In this position, F^−^ forms coordination bonds with three Mg^2+^ cations at distances (≤2 Å) consistent with previously observed Mg-F bond lengths (39). These bonds are slightly shorter than the coordination bonds formed by Mg^2+^ and water molecules, which are considered to be 2.1 Å long on average (40). The square planar coordination geometry of the F^−^ ion is completed not by a fourth coordination bond but rather by the γ-phosphorus atom of pppGpp, located 3.4 Å away. Some density peaks assigned to water molecules might instead be F^−^ ions as described in other structures (39,41–42); however, the lack of clear square planar coordination geometry precluded us from confidently making such assignments.

Superposition of the pppGpp- and RNA-bound structures (Figure 5D) revealed that Mg1, Mg2 and Mg3 are common to both structures while Mg4 was not observed in the RNA-bound structure (6). To cleave the bond between β- and α-phosphates of the RNA, RppH utilizes a water molecule located between Mg2 and Mg3 for in-line attack on the β-phosphorus (purple stick in Figure 5D). Although the pppGpp-bound structure contains the same water molecule, it is located too far from the substrate to attack. The structure raises a possibility that RppH uses an alternative mechanism to hydrolyze pppGpp. Analysis of the structure indicates that the F^−^ ion, positioned between Mg1 and Mg4, is located a short distance from the γ-phosphorus atom and forms an obtuse angle (123°) with this atom and the bridging oxygen atom. We posit that this F^−^ anion replaces a nucleophilic hydroxide ion or water molecule positioned for in-line attack (cyan stick in Figure 5B and D) on the γ-phosphorus atom after small conformational adjustments. Thus, the pppGpp-bound RppH structure may closely resemble the active conformation of RppH required for cleavage of the bond between the 5′ β- and γ-phosphates, using a nucleophile coordinated between Mg1 and Mg4.

### RppH uses the same mechanism to hydrolyze GTP and pppGpp

The structure of the RppH bound to GTP and Mg^2+^, determined at 2.35 Å resolution (Supplementary Table S2), revealed that GTP binding is very similar to the binding of the corresponding part of pppGpp (Figure 5E). Despite a slightly lower resolution, the electron density map was of sufficient quality to fit four Mg^2+^ cations, fluoride and a couple of Mg^2+^-coordinated water molecules in the catalytic site. Superposition with the pppGpp-bound structure revealed only minor conformational differences between bound GTP and pppGpp (Figure 5G), suggesting that RppH uses the same mechanism to cleave off the 5′ γ-phosphate from both substrates. By extrapolation, RppH likely hydrolyzes the 5′γ-phosphate of other NTPs in much the same way, by adding an extra Mg^2+^ ion to extend the catalytic site.

### GDP slides toward the active site for hydrolysis

GDP is too small for its terminal phosphate to reach even an extended catalytic site if its nucleobase binds to RppH in the same way as GTP. Instead, the structure of RppH bound to GDP and Mg^2+^, determined at 2.7 Å resolution (Supplementary Table S2), revealed that GDP slides toward the catalytic site by a distance that roughly corresponds to two phosphates of GTP (Figure 5F and G). As a result, the 5′ α-phosphate of GDP occupies the position of the 5′ γ-phosphate of GTP and maintains two of the corresponding intermolecular contacts with RppH. The β-phosphate of GDP moves deeper into the active site, where it replaces Mg4. Therefore, the catalytic site in the GDP-bound structure contains three Mg^2+^ cations in a configuration remarkably similar to that observed in the RNA-bound structure (Figure 5H). RppH likely catalyzes cleavage between the two phosphates of GDP by using a nucleophilic water molecule or hydroxide ion positioned between Mg2 and Mg3, as proposed for RNA substrates (6). In the crystal structure, this nucleophile is replaced by a fluoride ion located a short distance (3.2 Å) from the β-phosphorus atom, at a 157° angle compatible with in-line attack. In contrast to the second nucleobase of RNA, which inserts deeply into the protein cleft, insertion of the nucleobase of GDP is shallower and lacks some contacts formed by RppH-bound RNA.

### Removal or overproduction of RppH does not change the concentration of ATP in cells

**Figure 6.**
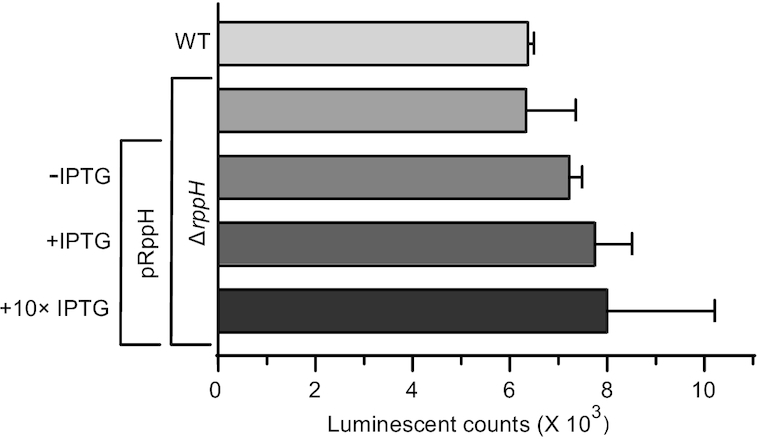
ATP content, assayed as luminescent counts (*n* = 3, mean ± SD), in *Escherichia coli* cells containing different amounts of RppH. Δ*rppH*, a strain from which the *rppH* gene has been deleted; pRppH, a plasmid for overproducing RppH upon induction with IPTG (10 μM) or 10 × IPTG (100 μM).

To determine whether hydrolysis by RppH noticeably affects the intracellular concentration of NTPs, we compared the concentration of ATP in wild-type *E. coli*, an isogenic Δ*rppH* strain and a Δ*rppH* strain containing a pPlacRppH plasmid for overproducing RppH (Figure 6). RppH overproduction was induced with 10 or 100 μM IPTG. The lower IPTG concentration resulted in RppH levels ∼250-fold greater than in wild-type cells (21), and we estimate that the higher IPTG concentration increased RppH levels by at least an additional factor of 4 (Supplementary Figure S6).

A luminescence assay, conducted on extracts of cultures grown to mid exponential phase, showed that the concentration of ATP was very similar in all three strains, irrespective of RppH levels (Figure 6). We conclude that nucleotide hydrolysis by this enzyme has little or no effect on the cellular concentration of ATP.

## DISCUSSION

In the current study, we have addressed an important question about the substrate specificity of the RNA-modifying enzyme RppH: how a key regulatory enzyme that converts tri- and diphosphorylated RNA 5′ termini to monophosphates is able to avoid depleting cells of NTPs, NDPs and other molecules that resemble RNA 5′ ends.

Our data show that, although NTPs, NDPs and the alarmone (p)ppGpp are hydrolyzed by RppH *in vitro*, they react more slowly than RNA substrates, especially in the pH range (7.1–7.8) typically maintained in *E. coli* cells (22,23). Moreover, measurements in wild-type cells, cells lacking RppH and cells overproducing RppH did not reveal significant differences in the concentration of ATP, evidence that RppH does not appreciably affect cellular NTP levels. This observation has dual explanation. RppH may not use ATP as a substrate effectively *in vivo* and/or *E. coli* can maintain stable ATP concentration (43) despite hydrolysis by RppH. At this time, we cannot distinguish between these two possibilities.

Nevertheless, the reaction of RppH with NTPs and other compounds accelerates at pH 8.0, a value close to the upper boundary of the pH level maintained in cells. These results suggest that under growth conditions that push the intracellular pH up to 8.1 (25), *E. coli* RppH may hydrolyze nucleotides at an increased rate. This problem could be aggravated in alkalophilic bacteria, such as the moderate halophile *Vibrio alginolyticus*, which possesses an RppH that is very similar in sequence to its *E. coli* counterpart but maintains a higher average intracellular pH of 7.8 (44). In contrast to nucleotide hydrolysis, our data indicate that the optimal pH for the reaction of RNA substrates, pH 7.5, matches the mean intracellular pH in *E. coli*. The difference in the optimal pH for the reaction of RppH with RNA and nucleotides is likely one of the factors that favor its reaction with RNA over other substrates.

**Figure 7.**
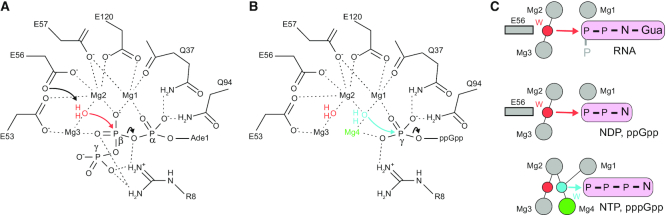
Proposed catalytic mechanisms of *Escherichia coli* RppH on various substrates. (**A**) Structure-based schematic of catalytic mechanism with the 5′-triphosphorylated RNA substrate (6). Reactive water molecule is in red. (**B**) Structure-based schematic of catalytic mechanism with pppGpp. An alternative reactive water molecule and an extra Mg^2+^ cation are shown in cyan and green, respectively. (**C**) Comparison of the different catalytic mechanisms derived from structural information. Substrates with 5′-terminal phosphates (p) are shown in pink rounded rectangles. N depicts a nucleoside. Mg^2+^ cations common to all structures are depicted as gray circles. Mg4, found in the pppGpp- and GTP-bound structures, is depicted in green circle. A reactive water for RNA (top panel) and NDP (middle panel) substrates is shown as red circle. A reactive water for pppGpp and NTP (bottom panel) substrates is shown as cyan circle. Arrows depict in-line attacks. Coordination of reactive water molecules by Mg^2+^ cations is depicted by black lines. A putative catalytic residue E56 is shown as gray rectangle.

Our published structures of *E. coli* RppH-Mg^2+^–RNA complexes (6) revealed specific binding of the second RNA nucleotide deep in the protein cleft. If nucleotides bind in the same cleft, their small size might be expected to prevent their 5′-terminal phosphates from reaching the catalytic site, a prediction at odds with their observed reactivity. The structures of pppGpp-, GTP- and GDP-bound RppH, crystallized in the presence of Mg^2+^ and F^−^ ions and trapped in the conformations poised for catalysis, resolved this apparent contradiction. These structures suggest that nucleotide hydrolysis may proceed through either of two different mechanisms (Figure 7). To remove the 5′ β-phosphate from GDP, ppGpp and likely other NDPs, RppH utilizes the catalytic mechanism previously described for the hydrolysis of RNA substrates (Figure 7A), involving three Mg^2+^ cations and a reactive water molecule coordinated by Mg2 and Mg3 (Figure 7C), but binds the nucleobase differently. In particular, the GDP-Mg^2+^-F^−^-RppH structure shows the nucleobase of mononucleotide substrates emerging from the deep cleft and moving toward the catalytic site, causing it to lose some interactions with the protein but enabling the 5′-terminal phosphates to reach the catalytic site. With GDP bound in this manner, a nucleophile is well situated for in-line attack on the β-phosphorus atom.

Other structures suggest an alternative mechanism to explain the cleavage of the 5′ γ-phosphate from pppGpp, GTP and likely other 5′-triphosphorylated nucleosides. In these structures, RppH reconfigures the catalytic site from that used for RNA and diphosphorylated nucleotides. The enzyme adds one more Mg^2+^ cation, Mg4, to the three Mg^2+^ cations previously observed in the RNA-bound structures (Figure 7B). This new cation, Mg4, is positioned closer to the substrate and, together with Mg1 and Mg2, coordinates a nucleophile for in-line attack. Thus, even though the nucleobase is bound deep in the protein cleft, an extra Mg^2+^ cation extends the catalytic site toward it and brings the nucleophile closer to the atom to be attacked (Figure 7C).

A similar arrangement with four Mg^2+^ cations next to a terminal phosphate was previously observed in structures of other Nudix proteins crystallized in the presence of F^−^ ions, for example, diphosphoinositol phosphatase 1 and MutT1 hydrolase (41,42). However, these structures differ in important ways from the RppH structure. The structures of the phosphatase and hydrolase were obtained in the presence of bound reaction products with the fourth Mg^2+^ cation imitating a phosphate of a substrate, reminiscent of the structures trapped by AlF_4_^−^ (40). In the RppH structure, the bound 5′-triphosphorylated nucleosides are reaction substrates.

If the hydrolysis of 5′-triphosphorylated nucleosides indeed involves a nucleophile sandwiched between Mg1 and Mg4, how closely does the high-resolution pppGpp-Mg^2+^-F^−^-RppH structure resemble the catalytically active conformation of the enzyme-substrate complex? Although F^−^ is located near the γ phosphorus atom, its angle of attack, 123°, is far from the 180° angle considered ideal for in-line attack. However, free energy simulations with ribozymes revealed that the angle of attack plays a modest role in enhancing the reaction rate, which remains acceptable even at a 145° angle (45). To approach a better angle for in-line attack, two small sterically possible shifts in the position of the nucleophile and the bridging oxygen atom are required. An adjustment in the position of the nucleophile is expected because Mg-O bonds are slightly longer than Mg-F bonds and because the coordination geometry of a water molecule would be tetrahedral rather than square planar.

The structures can also explain why nucleotide substrates require higher pH than RNA for efficient hydrolysis by RppH. Because the negatively charged oxygen on the hydroxide ion carries greater electron density than the oxygen atom of a neutral water molecule, a hydroxide ion is much more nucleophilic than a water molecule and reacts in an S_N_2 reaction faster than a water nucleophile (46). Although the rates of the nucleophilic attacks depend on the system, the difference between water and hydroxide ion could reach several orders of magnitude in reactions involving small organic molecules (47). At high pH, where the concentration of hydroxide ions is higher, they can more frequently occupy a position in the active site of RppH suitable for the nucleophilic attack on the phosphorus atom of substrates. However, a catalytically competent configuration of the substrate and metal cations may be more transient for substrates other than RNA. Indeed, in case of NTPs and pppGpp, catalysis depends on ligands that are more weakly bound: either Mg4, which does not bind RppH, or NDPs and ppGpp, which have fewer contacts with RppH than RNA. Therefore, the rate of the reaction with nucleotides should be especially dependent on high pH.

The structures also let us speculate as to why the hydrolysis of diphosphorylated nucleotide substrates, such as GDP, is faster than the hydrolysis of triphosphorylated substrates, such as GTP, at pH ∼8.0. GDP hydrolysis involves a nucleophile coordinated by Mg2 and Mg3. This nucleophile could be a water molecule activated by deprotonation. In *E. coli* RppH, Glu56 can reach the reactive water molecule and help to activate it (6). In the case of GTP, a nucleophile is positioned between Mg1, Mg2 and Mg4, where no RppH amino acid can activate it. Therefore, the preferred nucleophile must be a hydroxide ion, necessitating a shift to alkaline pH for optimal catalysis.

Our biochemical data demonstrate that nucleotides inhibit the reaction of RppH with RNA substrates. As suggested by the crystal structures, such inhibition could be caused by the competition of nucleotides with RNA for binding to the catalytic site and the nucleobase-binding cleft. Remarkably, nucleotides can bind in the cleft without anchoring their phosphates in the catalytic site by Mg^2+^-mediated interactions. Since nucleotides bind to RppH more weakly than RNA, rather high concentrations are required for them to inhibit the reaction of RNA. However, these inhibitory concentrations are comparable to intracellular concentrations of NTPs (4,5). Published data have shown that intracellular concentrations of NTPs and NDPs range from ∼0.3 to 10 mM and from ∼0.1 to 1.8 mM, respectively (4,5). Specifically, GTP and GDP concentrations are ∼0.7–1.2 mM and ∼ 0.1–0.2 mM, respectively, depending on the growth phase (5). Higher values, ∼4.9 and ∼0.7 mM, were determined for GTP and GDP in an independent study (4). Thus, the inhibitory concentration of GDP (IC_50_ ∼30–60 μM) is slightly below its intracellular concentration, while that of GTP (IC_50_ ∼1.6–4.1 mM) is at the high end of its intracellular concentration.

ppGpp concentrations can increase from 40 μM during exponential growth to 0.8 mM during transition to stationary phase, while during the stringent response ppGpp becomes the most abundant nucleotide, accounting for 60% of the cellular nucleotide pool (5). pppGpp, which is undetectable in untreated cultures, increases to ∼8% of the nucleotide pool during the stringent response, a concentration slightly higher than that of GDP under these conditions (5). Our IC_50_ measurements (50–200 μM) suggest that ppGpp and pppGpp can strongly inhibit the reaction of RppH with mRNA substrates under stresses that cause the concentration of (p)ppGpp to surge. Consequently, an RppH-mediated pathway for 5′-end-dependent RNA degradation may become less efficient under these stresses. Such a change may increase the stability of hundreds of mRNAs whose degradation is RppH-dependent.

Interestingly, about 2-3-fold higher concentrations of nucleotides are needed to inhibit the reaction of RppH with diphosphorylated RNA substrates than with the triphosphorylated RNA. Therefore, the more efficient competition of nucleotides with triphosphorylated RNA augments its intrinsically lower reactivity with RppH (3) in determining the preference of RppH for diphosphorylated RNA substrates *in vivo*. Thus, a competition between cognate and non-cognate substrates likely shapes the specificity of this enzyme in bacterial cells.

In summary, these studies suggest that *E. coli* RppH tunes gene expression by integrating two types of signals that affect the reactivity and specificity of the enzyme. In addition to stimulation of RppH by DapF binding, as previously reported (7–9), RppH likely experiences inhibition in cells by various small molecules that serve as non-cognate substrates and competitors of RNA substrates. While some of these compounds, such as NTPs and NDPs, are always present in cells and constantly inhibit RppH, other compounds, such as pppGpp and ppGpp, most dramatically affect RppH activity only under certain growth conditions that maximize their biosynthesis. The impact of these signals on RppH activity enables the differential control of RNA degradation in a variety of RppH-associated cellular processes important for the adaptation of bacteria to diverse growth conditions encountered during stress (28), host invasion (27,31,48–49) and host–pathogen interactions (50).

## DATA AVAILABILITY

Coordinates of the structures were deposited to the Protein Data Bank with PDB ID codes: 6VCQ, GTP–RppH complex; 6VCN, ppcpG–RppH complex; 6VCO, ppcpA–RppH complex; 6VCR, CTP–RppH complex; 6VCP, UTP–RppH complex; 6VCL, pppGpp-Mg^2+^-F^−^–RppH-DapF complex; 6VCM, GTP-Mg^2+^-F^−^–RppH-DapF complex; 6VCK, GDP-Mg^2+^-F^−^–RppH-DapF complex. All relevant data and materials are available upon request.

## Supplementary Material

gkaa024_Supplemental_File
