# Supplementary material for: Principles of RNA and nucleotide discrimination by the RNA processing enzyme RppH
Source: Nucleic Acids Res. 2020 Jan 21;48(7):3776–88. doi: 10.1093/nar/gkaa024 (PMC7144940; doi:10.1093/nar/gkaa024)
Supplement: gkaa024_Supplemental_File [file gkaa024_supplemental_file.pdf]

Supplementary Information

Principles of RNA and nucleotide discrimination by the RNA processing enzyme RppH

Ang Gao[#], Nikita Vasilyev[#], Abhishek Kaushik, Wenqian Duan and Alexander Serganov

[#] These authors contributed equally

Department of Biochemistry and Molecular Pharmacology,

New York University School of Medicine, 550 First Avenue, New York, NY 10016, USA

Table S1. Data collection and refinement statistics for RppH-NTP complexes

Dataset	ppcpG	ppcpA	CTP	UTP	GTP
**Data collection**					
Wavelength	1.5418	1.5418	1.5418	1.5418	1.5418
Space group	$P3_121$	$C2$	$C2$	$C2$	$P3_121$
Unit Cell					
a, b, c (Å)	61.8, 61.8, 72.3	79.1, 39.0, 57.7	78.5, 38.8, 57.7	78.8, 38.7, 58.8	60.7, 60.7, 74.3
Resolution (Å)	19.48-1.90 (1.97- 1.90)[a]	19.48-1.70 (1.76- 1.70)	19.31-1.60 (1.66-1.60)	19.37-1.70 (1.76-1.70)	19.18-1.60 (1.7-1.6)
R_{merge} [b]	0.090 (0.672)	0.047 (0.472)	0.035 (0.341)	0.057 (0.542)	0.062 (0.467)
$CC_{1/2}$	0.998 (0.718)	0.999 (0.923)	0.999 (0.887)	0.999 (0.774)	0.999 (0.672)
$I/\sigma(I)$	16.4 (2.7)	24.0 (4.0)	23.5 (4.0)	15.6 (2.3)	14.4 (2.2)
Completeness (%)	99.6 (98.5)	95.3 (89.6)	99.0 (92.9)	99.2 (96.6)	99.1 (95.2)
Redundancy	6.1 (5.9)	7.1 (7.0)	4.5 (3.8)	3.9 (3.8)	3.8 (2.5)
No. of unique reflections	12,944 (1,244)	18,406 (1,706)	22,689 (2,121)	19,245 (1,896)	39,944 (6,148)
**Refinement**					
Resolution (Å)	19.48-1.90	19.48-1.70	19.31-1.60	19.37-1.70	19.18-1.60
R_{work}/R_{free} (%)	20.0/25.2	17.2/20.3	16.4/19.0	18.2/20.8	17.3/20.6
No. of atoms					
Protein	1,304	1,311	1,302	1,286	1,312
Ligand	32	31	92	151	32
Water	117	153	186	58	168
Ion	20	-	5	-	25
Average B factor (Å^2)					
Protein	26.2	30.8	23.2	24.2	18.3
Ligand	43.2	43.3	43.5	37.2	36.9
Water	29.7	35.9	31.6	31.4	26.7
Ion	26.0	-	34.6	-	27.7
R.m.s. deviations					
Bond lengths (Å)	0.013	0.012	0.008	0.011	0.006
Bond angles (°)	1.29	1.19	1.34	1.21	0.94
Ramachandran analysis					
Favored (%)	96.1	98.7	100.0	98.7	98.1
Outliers (%)	0	0	0	0	0
Estimated error [c]	0.25	0.18	0.14	0.16	0.15

[a] Highest resolution shell (in Å) shown in parentheses.
[b] $R\text{merge}=\sum_{hkl}\sum_{i=1}^{n}|Ii(hkl)-\bar{I}(hkl)|/\sum_{hkl}\sum_{i=1}^{n}Ii(hkl)$, where Ii (*hkl*) is the *i*th observation of reflection *hkl* and $\bar{I}$(*hkl*) is the weighted average intensity for all *i* observations of reflection *hkl*.
[c] Estimated coordinate error based on maximum likelihood was calculated by Phenix.refine.

Table S2. Data Collection and Refinement Statistics for RppH-DapF-NTP complexes

Dataset	GTP	GDP	pppGpp
**Data collection**			
Wavelength	0.9791	0.9791	0.9793
Space group	$C222_1$	$C222_1$	$C222_1$
Unit Cell			
a, b, c (Å)	162.4, 192.9, 51.1	161.8, 193.6, 51.0	162.4, 190.6, 51.3
Resolution (Å)	30.00-2.35 (2.40-2.35)[a]	30.00-2.70 (2.8-2.7)	30.00-2.05 (2.09-2.05)
R_{merge} [b]	0.090 (1.093)	0.081 (1.139)	0.113 (0.819)
$CC_{1/2}$	0.994 (0.650)	0.995 (0.698)	0.996 (0.607)
$I/\sigma(I)$	22.2 (1.5)	24.6 (1.5)	21.2 (1.3)
Completeness (%)	99.9 (100.0)	99.9 (100.0)	98.2 (94.0)
Redundancy	6.6 (6.7)	6.6 (6.7)	6.8 (4.4)
No. of unique reflections	33,797 (2,208)	22,657 (2,203)	48,690 (2,296)
**Refinement**			
Resolution (Å)	29.9-2.35	28.93-2.69	30.08-2.06
R_{work}/R_{free} (%)	21.2/23.4	22.6/26.8	19.1/22.7
No. of atoms			
Protein	3,440	3,431	3,436
Ligand	32	28	40
Water	73	102	280
Ion	6	5	7
Average B factor (Å^2)			
Protein	61.2	80.1	45.6
Ligand	75.0	114.8	59.2
Water	55.6	76.8	49.8
Ion	72.2	86.4	37.7
R.m.s. deviations			
Bond lengths (Å)	0.003	0.005	0.008
Bond angles (°)	0.69	0.78	1.01
Ramachandran analysis			
Favored (%)	97.7	94.9	97.7
Outliers (%)	0	0	0
Estimated error [c]	0.25	0.38	0.25

[a] Highest resolution shell (in Å) shown in parentheses.

[b] $R\text{merge}=\sum_{hkl}\sum_{i=1}^{n}|Ii(hkl)-\bar{I}(hkl)|/\sum_{hkl}\sum_{i=1}^{n}Ii(hkl)$, where Ii (*hkl*) is the *i*th observation of reflection *hkl* and $\bar{I}$(*hkl*) is the weighted average intensity for all *i* observations of reflection *hkl*.

[c] Estimated coordinate error based on maximum likelihood was calculated by Phenix.refine.

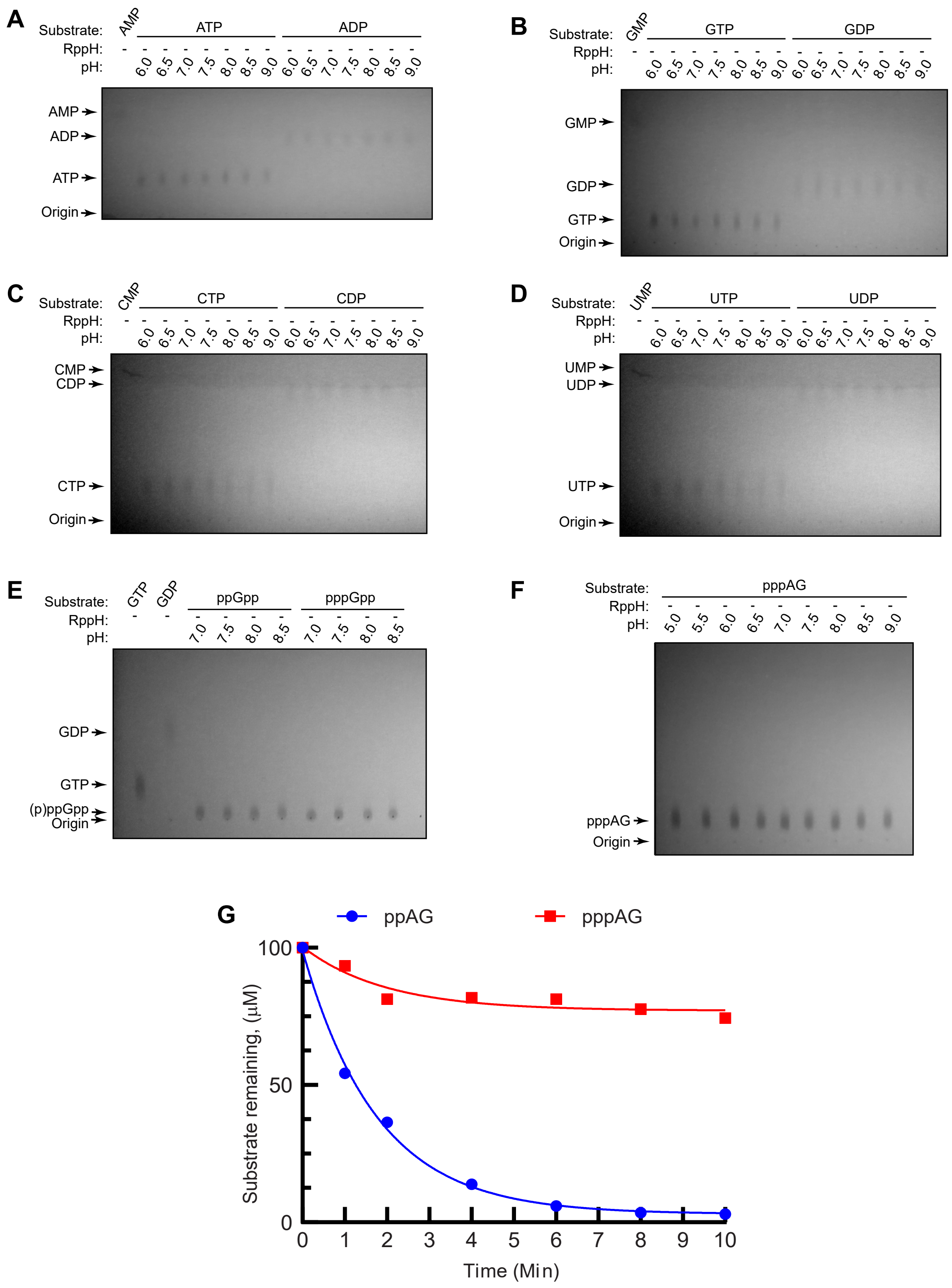

Figure S1. Hydrolysis of the 5′-phosphorylated ends of nucleotides and RNA. (**A-F**), Removal of 5′ phosphates from ATP and ADP (A), GTP and GDP (B), CTP and CDP (C), UTP and UDP (D), ppGpp and pppGpp (E), and pppAG RNA (F) at different pHs in the absense of RppH. The cleavage products were analyzed by TLC. (**G**), Kinetics of hydrolysis of the 5′-diphosphate and 5′-triphosphate of the RNA substrates ppAG and pppAG, respectively, by *E. coli* RppH. Reaction products and substrates were separated by ion-exchange chomatography, and cleavage of the 5′ phosphates was fitted to a first order model by using GraphPad. The initial reaction rates, determined from the first four time points (0-4 min), were 3.3 ± 2.3 and 24.1 ± 2.3 µM/min for pppAG and ppAG, respectively. Note that the RppH concentration was only one-tenth of that in similar experiments with nucleotides.

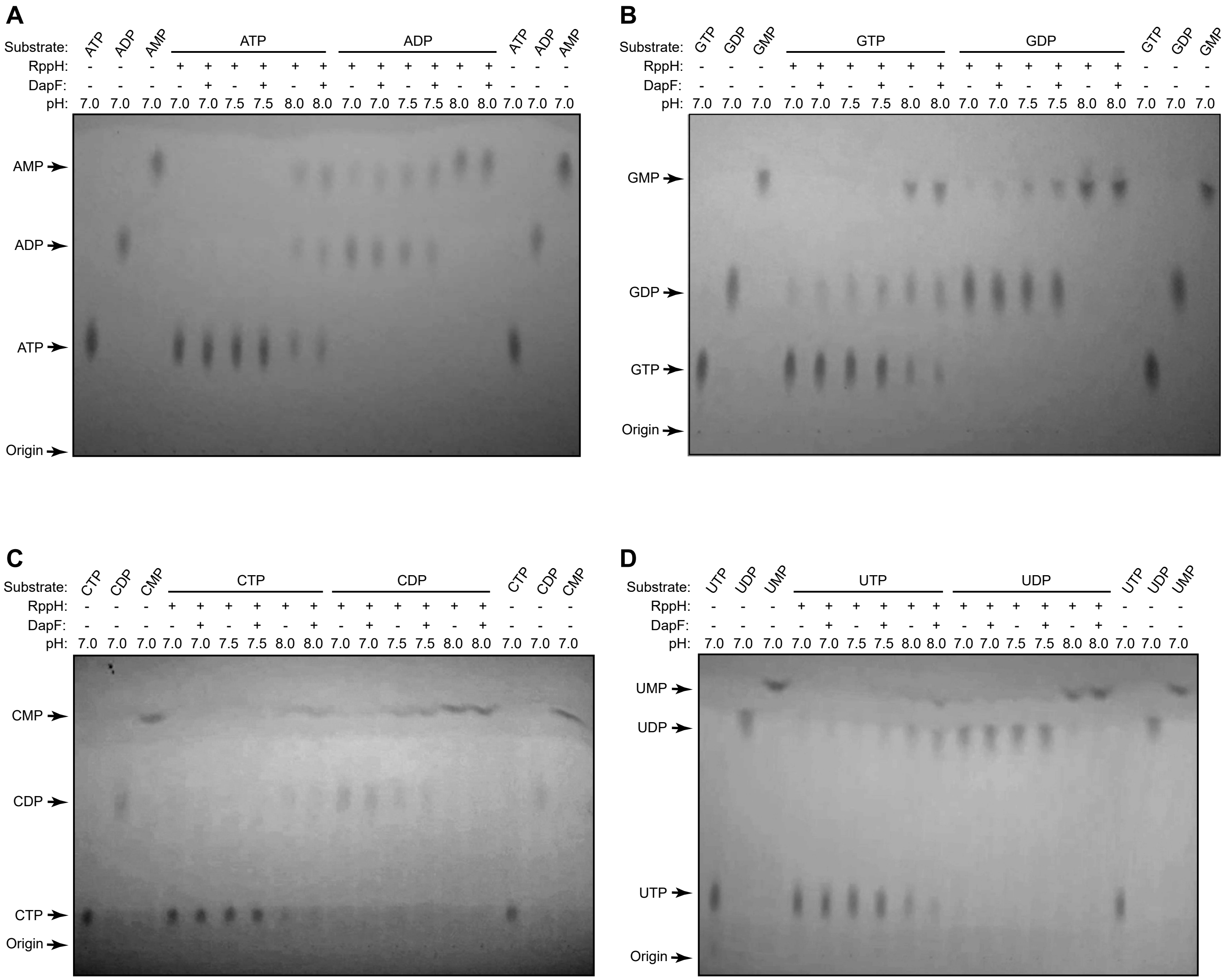

Figure S2. Hydrolysis of the 5′-triphosphates and 5′-diphosphates of NTPs and NDPs by *E. coli* RppH at various pHs, in the presence or absence of *E. coli* DapF. ATP and ADP (**A**), GTP and GDP (**B**), CTP and CDP (**C**), and UTP and UDP (**D**). The cleavage products were analyzed by TLC.

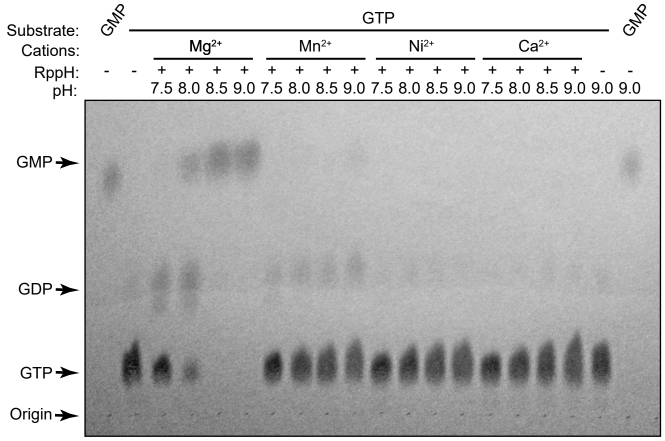

Figure S3. Metal ion dependence of the reactivity of RppH with GTP. Reactions in the presence of various divalent cations at pH 7.5-9.0 were analyzed by TLC. Mg^{2+} cations support hydrolysis most efficiently, while Mn^{2+} cations stimulate catalysis with very low efficiency and Ni^{2+} and Ca^{2+} cations do not support hydrolysis.

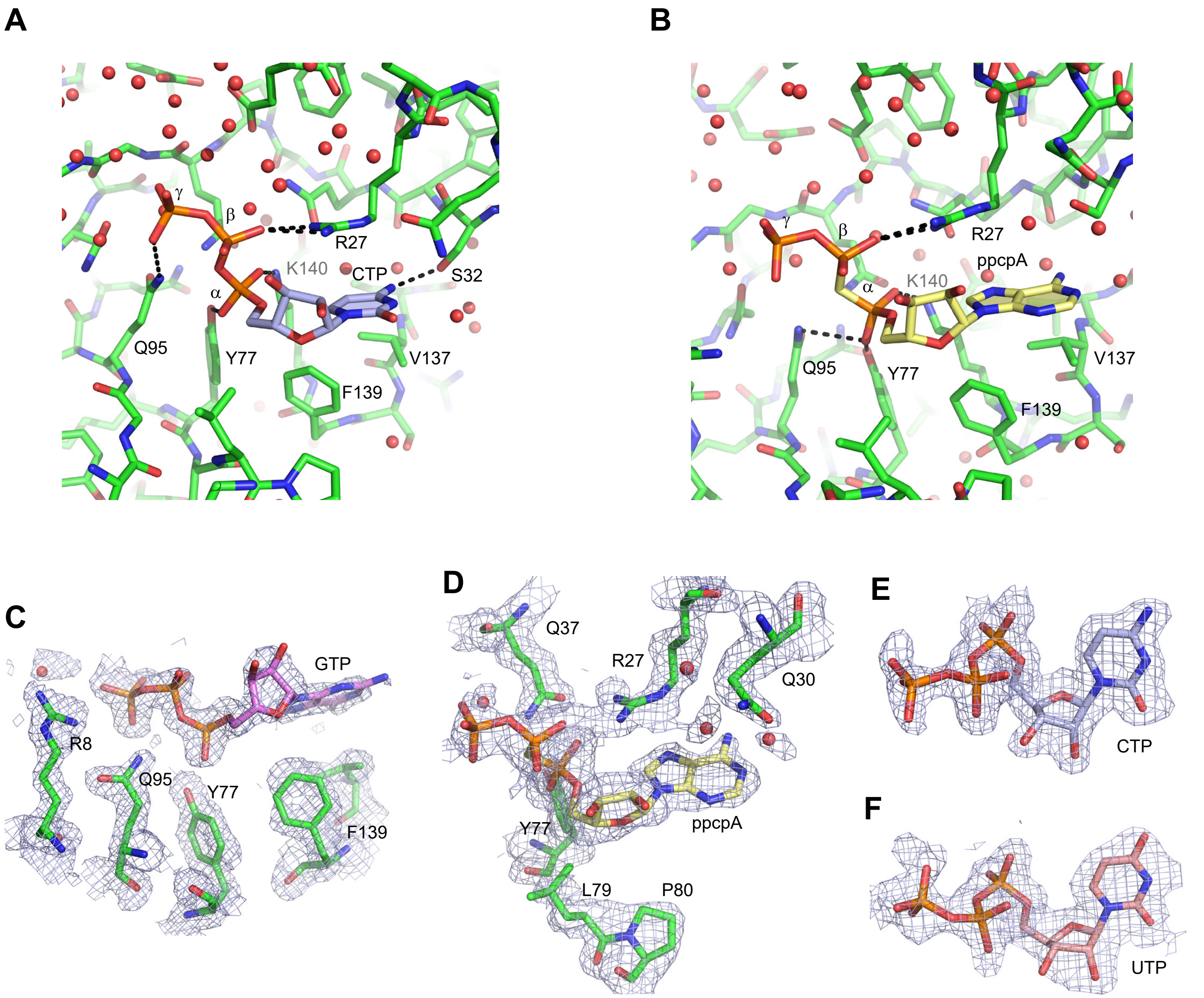

Figure S4. Crystal structures of NTPs bound to *E. coli* RppH. (**A** and **B**), Zoomed-in views of CTP- (light blue) and ppcpA-bound (yellow) RppH structures. RppH is shown in green sticks. Putative hydrogen bonds are shown as dashed lines. Water molecules are red spheres. Phosphates are indicated with Greek characters. (**C**-**F**), Composite simulated annealing omit map (gray mesh, 1 σ level) shown with the refined GTP-RppH (C), ppcpA-RppH (D), CTP-RppH (E), and UTP-RppH (F) structures.

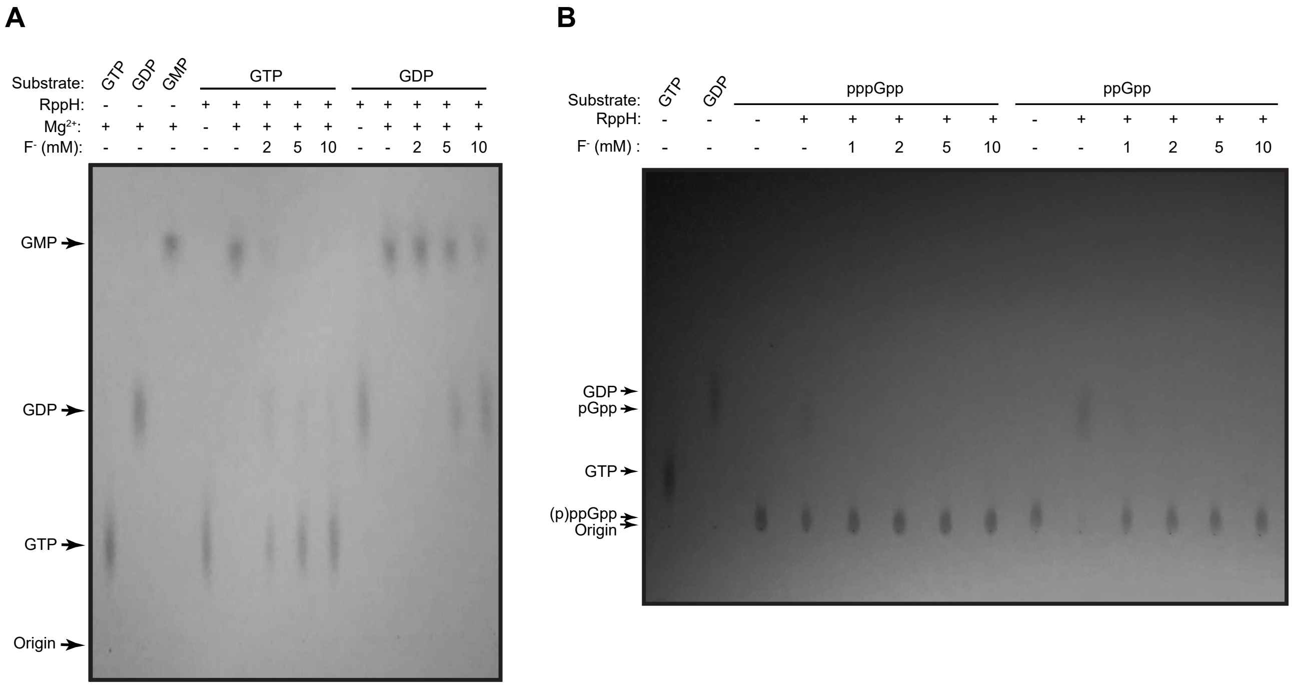

Figure S5. Inhibition of *E. coli* RppH activity by fluoride (F^-). Reaction products were analyzed by TLC. (**A**), Reactions with GTP and GDP at pH 8.0. (**B**), Reactions with pppGpp and ppGpp at pH 7.5.

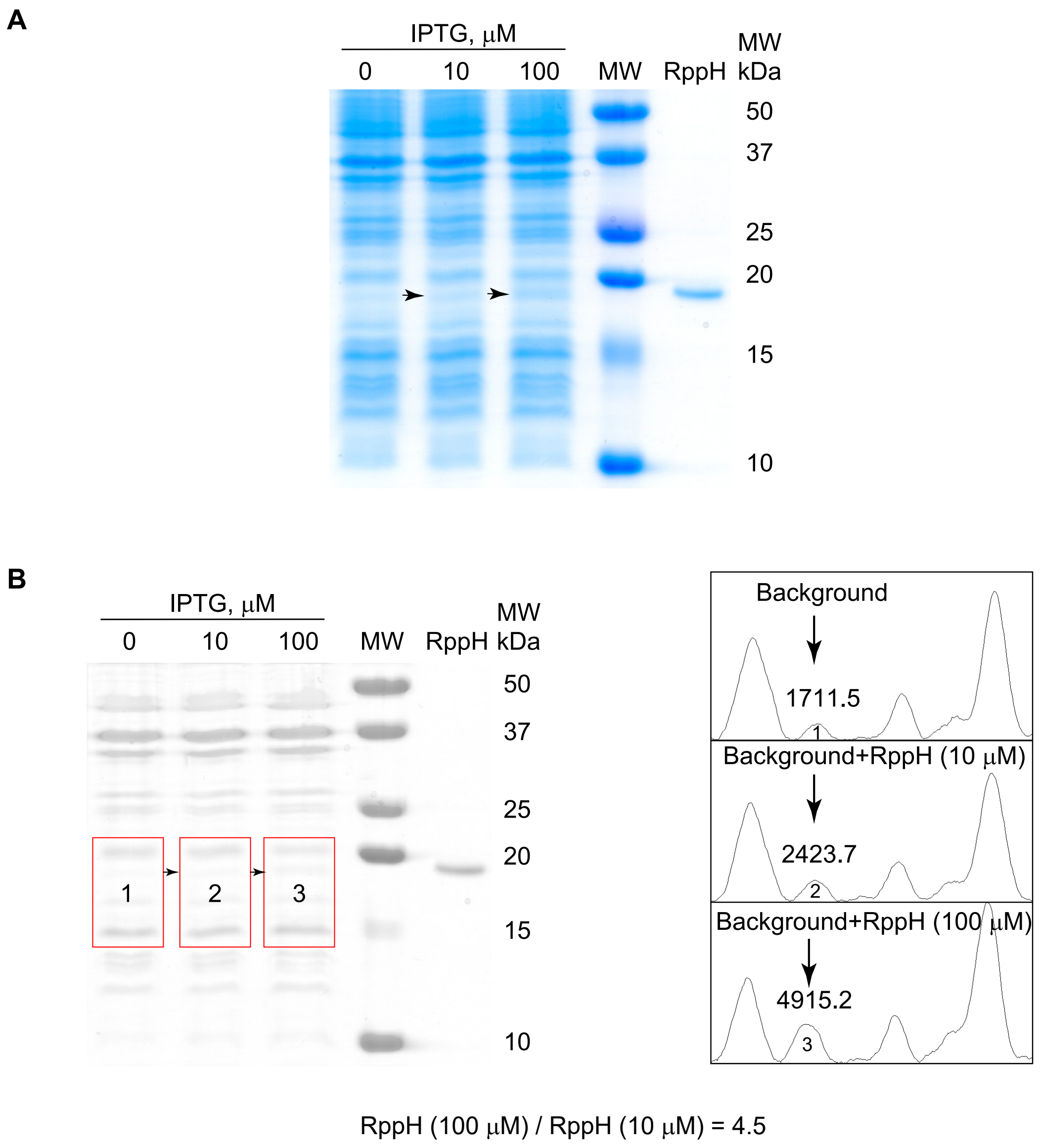

Figure S6. Overproduction of *E. coli* RppH in an *E. coli* ΔrppH strain containing plasmid pPlacRppH. (**A**), Stained SDS gel showing the production of RppH (arrows). (**B**), Quantification of RppH levels. Left, gel image quantified by Image J. Red rectangles show regions used for quantification. Right, profiles depicting the density of protein bands in the red rectangles. The peaks corresponding to the region where RppH migrates are numbered. RppH production is ~4.5 fold higher at 100 μM than at 10 μM IPTG.
